# Development of Syringaldehyde as an Agonist of the GLP-1 Receptor to Alleviate Diabetic Disorders in Animal Models

**DOI:** 10.3390/ph17040538

**Published:** 2024-04-22

**Authors:** Jenpei Lee, Yingxiao Li, Juei-Tang Cheng, I-Min Liu, Kai-Chun Cheng

**Affiliations:** 1 Department of Neurosurgery, Da Chien General Hospital, Miaoli City 36052, Taiwan; jenpeilee@yahoo.com.tw; 2 Department of Nursing, Tzu Chi University of Science and Technology, Hualien City 970302, Taiwan; yxli@ems.tcust.edu.tw; 3 Graduate Institute of Medical Science, Chang Jung Christian University, Tainan City 71101, Taiwan; 4 Department of Pharmacy, College of Pharmacy and Health Care, Tajen University, Pingtung 90741, Taiwan; iml@tajen.edu.tw

**Keywords:** syringaldehyde, liraglutide, GLP-1 receptor, inflammatory cytokines, complications, diabetic rats

## Abstract

The phenolic aldehyde syringaldehyde (SA) has been shown to have an antihyperglycemic effect in diabetic rats due to increased glucose utilization and insulin sensitivity. To understand the direct effect of SA on the GLP-1 receptor, STZ-induced diabetic rats were used. The levels of pro-inflammatory cytokines, liver enzymes, and renal function were measured using specific ELISA kits. The mechanisms of SA effects were investigated using CHO-K1 cells, pancreatic Min-6 cells, and cardiomyocyte H9c2 cells. The results indicated that the antihyperglycemic effect of SA in diabetic rats was abolished by blocking the GLP-1 receptor with an antagonist. SA has a direct effect on the GLP-1 receptor when using CHO-K1 cells transfected with the exogenous GLP-1 receptor gene. In addition, SA stimulated insulin production in Min-6 cells by activating GLP-1 receptors. SA caused a dose-dependent rise in GLP-1 receptor mRNA levels in cardiac H9c2 cells. These in vitro results support the notion that SA has a direct effect on the GLP-1 receptor. Otherwise, SA inhibited the increase of pro-inflammatory cytokines, including interleukins and tumor TNF-α, in type 1 diabetic rats in a dose-dependent manner. Moreover, as with liraglutide, SA reduced plasma lipid profiles, including total cholesterol and triglyceride, in mixed diet-induced type 2 diabetic rats. Intriguingly, chronic treatment with SA (as with liraglutide) reversed the functions of both the liver and the kidney in these diabetic rats. SA displayed less efficiency in reducing body weight and food consumption compared to liraglutide. In conclusion, SA effectively activates GLP-1 receptors, resulting in a reduction in diabetic-related complications in rats. Therefore, it is beneficial to develop SA as a chemical agonist for clinical applications in the future.

## 1. Introduction

In clinics, ‘Incretin-Based Therapy’ is widely used to treat metabolic disorders and diabetic complications [1]. After consuming a meal, there is an increase in the release of incretins and insulin [2]. Both incretins, glucose-dependent insulinotropic peptide (GIP) and glucagon-like peptide-1 (GLP-1), have short half-lives of around 1–2 min due to the fast degradation caused by the endogenous enzyme dipeptidyl peptidase 4 (DPP-4), which significantly diminishes their effectiveness [3]. DPP-4 inhibitors inhibit the inactivation of both GIP and GLP-1, thereby elevating GLP-1 and GIP levels, though the magnitude of elevation is small (picomolar) when compared with analog supplementation (nanomolar) during the activation of the receptor site [4]. DPP-4 inhibitors used in clinics include sitagliptin, saxagliptin, linagliptin, and alogliptin. Each can be used alone or in combination with other medications such as metformin, empagliflozin, or pioglitazone. DPP-4 inhibitors have been shown to reduce albuminuria as well as lower blood glucose [5].

Incretin-based therapy has already been studied using DPP-4 inhibitors and GLP-1 analogs over prolonged circulation times [1]. GLP-1 analogs for clinical use include the short-acting agonists Exenatide and Lixisenatide, which have a half-life (t 1/2) of less than 3 h, and they should be administered twice daily. DPP-4-insensitive long-acting agonists, such as Liraglutide, Dulaglutide, and Semaglutide, were developed for widespread use. GLP-1 receptor agonists belong to the peptide analogs that should be administered by means of injection. Semaglutide is the only agonist available in both injectable and oral formulations [6]. Recently, these agonists have been utilized in body weight reduction, with clinical reports noting that they contribute 0.1 kg to the weight loss superiority of semaglutide [7] compared with other agonists (dulaglutide or liraglutide). Tirzepatide is the first ‘twincretin,’ meaning it is an agonist for both GLP-1 and GIP receptors [8]. It is a 39-amino acid synthetic peptide that can be injected subcutaneously once a week. Tirzepatide demonstrated impressive glycemic efficacy and weight loss over a year of use in six controlled trials [9]. Therefore, tirzepatide may be developed for the treatment of obese people in the future.

Natural products are potential candidates for the creation of novel compounds for diabetic therapy [10]. Several natural compounds, including d-allulose and glycylsarcosine, have been shown to enhance GLP-1 expression and stimulate insulin secretion [11]. Some food ingredients, including ginseng, ginger, glutamine, garlic, and monounsaturated fatty acids, have been shown to increase GLP-1 levels [12]. These products have been associated with an increase in GLP-1 secretion [13]. Conversely, several natural substances also directly stimulate the GLP-1 receptors. These substances include Geniposide [14], Catalpol [15], Puerarin [16], Myricetin [17], Morroniside [18], and Cinchonine [19].

Syringaldehyde (SA), otherwise known as 3,5-dimethoxy-4-hydroxybenzaldehyde, is an active component that is isolated from the stems of *Hibiscus taiwanensis* S. Y. Hu (Malvaceae); it has been shown to have an antihyperglycemic effect in diabetic rats [20]. In addition, enhanced glucose utilization and improved insulin sensitivity are the potential mechanisms generated by SA that are likely to be responsible for lowering blood glucose levels [15]. In silico investigation indicates that there is a correlation between SA and the GLP-1 receptor [21], as discussed in a previous review [22]. Therefore, understanding the direct effect of SA on the GLP-1 receptor is the main aim in the current report. Additionally, the effects of SA on diabetic complications were also investigated in two kinds of animal models with diabetes.

## 2. Results

### 2.1. Activation of the GLP-1 Receptor Using SA

**Figure 1:**
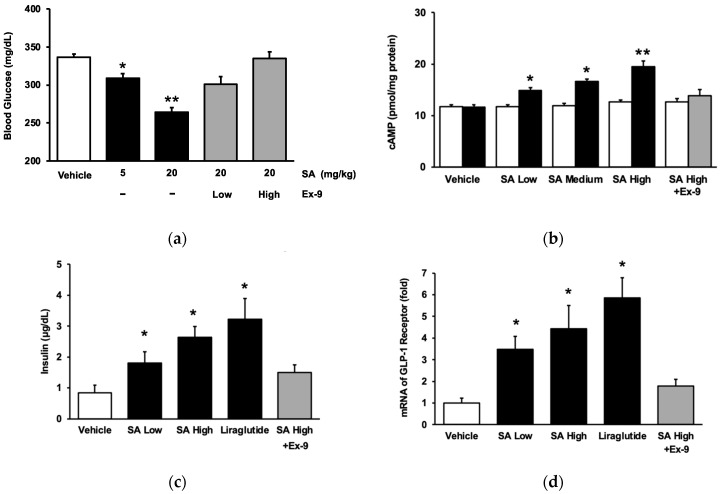
The role of syringaldehyde (SA) on glucose metabolism via activating GLP-1 receptors. (**a**) The reduction in blood glucose levels after an intravenous administration of SA at either a low dosage (5 mg/kg) or a high dosage (20 mg/kg) was dependent on the dose. Exendin 9-39 (Ex-9) was administered intraperitoneally at a low dosage of 0.05 mg/kg or a high dose of 0.1 mg/kg to effectively block the GLP-1 receptor. (**b**) SA increased cAMP levels in CHO-K1 cells transfected with the GLP-1 receptor (black column) as compared with the group treated with the vehicle (open column). An increase in cAMP levels was observed at concentrations of 0.1 μM (low), 0.5 μM (medium), and 1 μM (high). (**c**) SA increased insulin secretion in Min-6 cells. (**d**) SA (incubated for 1 h) increased the mRNA levels of GLP-1 receptor in H9c2 cells. Ex-9, at a concentration of 0.5 μM, was pre-incubated for 30 min prior to treatment. The value of each indicator is shown in a column as the mean ± standard error of the mean (SEM) per group; the sample size was eight. * The statistical significance (*p* < 0.05) and ** The statistical significance (*p* < 0.01) was obtained by comparing the treated group with the group treated with the vehicle only.

In line with our earlier study [20], SA demonstrated a reduction in hyperglycemia in rats with streptozotocin (STZ)-induced diabetes, as seen in Figure 1a. Subsequently, Ex-9, a GLP-1 receptor antagonist, was administered to diabetic rats to inhibit the receptor, following the previously published protocol [23]. Ex-9 exhibited a dose-dependent reversal of the inhibitory action of SA, as seen in Figure 1a. Ultimately, Ex-9 counteracted the impact of SA at a dosage that could effectively eliminate the influence of liraglutide. The impact of SA on the GLP-1 receptor was also determined in cultured cells. We used CHO-K1 cells for the transfection of the exogenous GLP-1 receptor gene, following the methodology outlined in a prior publication [24]. SA caused a considerable increase in intracellular cAMP content in a dose-dependent manner in the CHO cells expressing the GLP-1 receptor (Figure 1b). SA did not affect the cAMP level in CHO-K1 cells that did not express GLP-1 (Figure 1b). The increase in cAMP concentration, which was observed in cells transfected with the GLP-1 receptor and caused by SA, was attributed to the activation of GLP-1 receptors when compared with the control group. Furthermore, we used Min-6 cells to ascertain the direct impact of SA on the GLP-1 receptor during insulin secretion in a laboratory setting. The role of SA in insulin secretion was investigated using Min-6 cells. In line with a previous work involving diabetic mice [25], it was shown that SA increased the secretion of insulin from pancreatic cells, in a manner that depended on the dosage, during exposure to a glucose concentration of 15 mmol/L (Figure 1c). Interestingly, Ex-9 significantly inhibited the GLP-1 receptor at a concentration of 100 nmol/L. Thus, SA stimulated the GLP-1 receptor to augment insulin release from pancreatic cells, a fact that has not been previously stated. Furthermore, we replicated our prior approach [23] to validate the impact of SA on the GLP-1 receptor in cardiomyocytes. Same as liraglutide, the mRNA levels of the GLP-1 receptor in H9c2 cells were elevated by SA in a dose-related manner and blocked by Ex-9 (Figure 1d). 

### 2.2. Inhibitory Effects of SA on Inflammation in Animals

**Figure 2:**
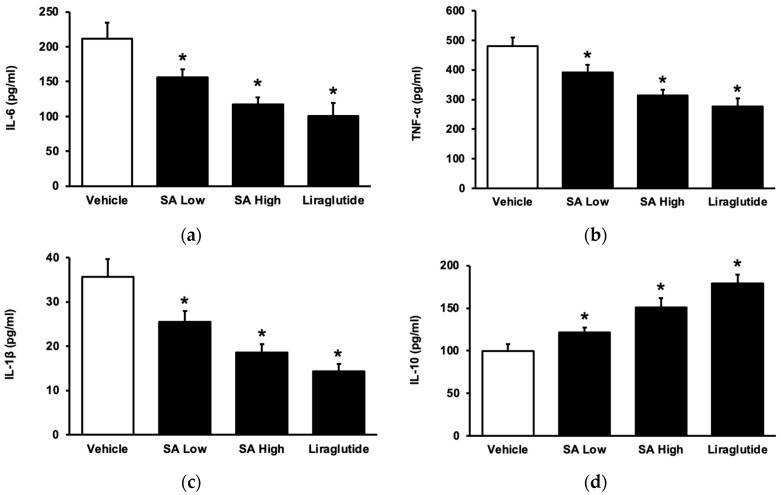
SA alters the levels of circulating cytokines in rats with type 1 diabetes. (**a**) The level of plasma interleukin-6 (IL-6) was reduced, in a dose-dependent manner, by utilizing liraglutide as a reference. (**b**) SA reduced the plasma level of tumor necrosis factor-α (TNF-α) in diabetic rats. (**c**) SA decreased the plasma level of IL-1β in diabetic rats. (**d**) SA increased the levels of the anti-inflammatory cytokine interleukin 10 (IL-10) in diabetic rats. The value of each indicator is shown in a column as the mean ± standard error of the mean per group (*n* = 8). * The statistical significance (*p* < 0.05) was obtained by comparing the treatment group with the diabetes group treated with the vehicle.

To understand the role of SA in inflammation in the advancement of diabetes mellitus [26], STZ-induced diabetic rats were administered with SA for a duration of one week [27]. SA dose-dependently reduced the elevated levels of plasma cytokines, including interleukin-6 (IL-6) (Figure 2a), tumor necrosis factor-α (TNF-α) (Figure 2b), and interleukin (IL-1β) (Figure 2c). This effect was the same as that observed with liraglutide. SA also increased anti-inflammatory cytokine (IL-10) levels (Figure 2d). Indeed, SA may inhibit inflammatory cytokines, similarly to liraglutide, and it has been identified in type 1 diabetic rats.

### 2.3. Inhibitory Effects of SA on Lipid Profiles and Hepatic Disorders in Animals

**Figure 3:**
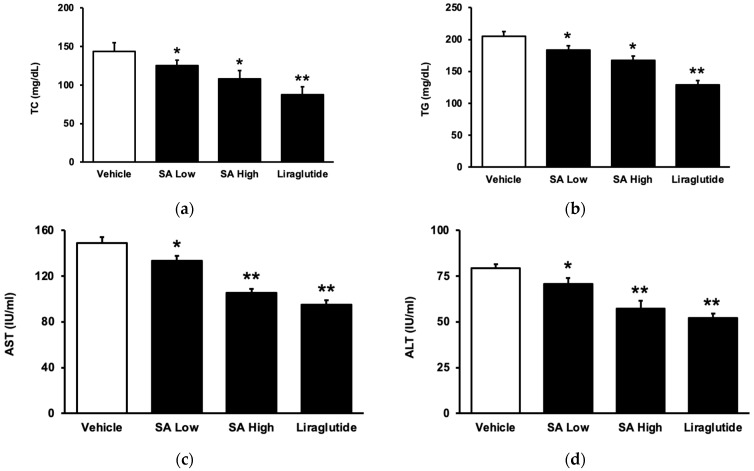
Circulating lipid levels and liver biomarkers modified by SA in type 2 diabetic rats. Rats with type 2 diabetes mellitus (T2DM) were given SA orally once a day in two doses (5 mg/kg as a low dose and 20 mg/kg as a high dose). In addition, another group received an intraperitoneal injection of liraglutide (0.2 mg/kg) for 8 weeks. (**a**) The plasma total cholesterol (TC) level was reduced by SA. (**b**) The plasma triglyceride (TG) level was also attenuated by SA. The levels of the liver enzymes, (**c**) aspartate aminotransferase (AST), and (**d**) alanine aminotransferase (ALT) induced by SA, were compared. The value of each indicator is displayed in a column as the mean SEM per group (*n* = 8). * *p* < 0.05 vs. vehicle-treated diabetic group; ** *p* < 0.01 vs. vehicle-treated diabetic group.

SA may attenuate hyperlipidemia in diet-induced diabetic rats after chronic treatment, which was similar to that shown in liraglutide. As shown in Figure 3a, the total cholesterol (TC) level was dose-dependently reduced with SA. Additionally, the plasma triglyceride (TG) level also decreased in the same manner (Figure 3b). As in a previous report [27], plasma aspartate aminotransferase (AST) activity markedly increased in these diet-induced diabetic rats (Figure 3c). Moreover, an increase in alanine aminotransferase (ALT) (Figure 3d), another hepatic biomarker [28], was also found in the serum of these rats. Persistent therapy with SA effectively and dose-dependently reversed both alterations. 

### 2.4. SA Improved Diabetic Nephropathy but Not the Body Weight in Animals

**Figure 4:**
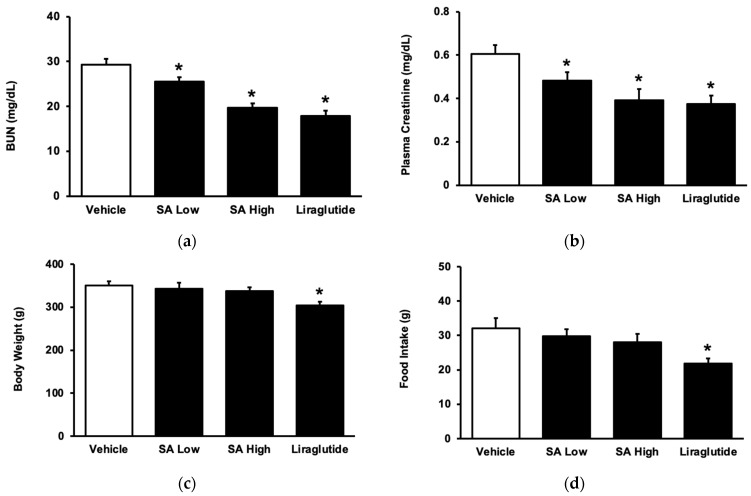
The renal indicators, body weight, and food intake of rats with T2DM are compared after long-term SA treatment. T2DM rats were administered with SA orally once daily at two different doses: 5 mg/kg as a low dosage and 20 mg/kg as a high dosage for 8 weeks. The rats received liraglutide (0.2 mg/kg/day, i.p.) as a positive control. Continuous treatment of SA may decrease both (**a**) the BUN level and (**b**) the blood creatinine level. The body weight (**c**) and food intake (**d**) were also compared following long-term treatment with SA in rats with T2DM. The value of each indicator is shown in a column as the mean ± standard error of the mean (SEM) per group; the sample size was eight. * *p* < 0.05 vs. the vehicle-treated diabetic group.

This study evaluated the effects of SA on diabetic nephropathy by measuring blood urea nitrogen (BUN) and serum creatinine levels. Figure 4a shows that there was a significant increase in BUN levels in rats that were given a high-fat diet (HFD), which was subsequently reversed by using SA in a dose-dependent manner. The same changes in the serum creatinine of these rats were also observed in these rats when SA was used (Figure 4b). Therefore, SA can alleviate the diabetic nephropathy induced by an HFD in rats. Additionally, treatment with liraglutide is known to combat obesity [29]. However, SA was less effective than liraglutide in terms of its ability to reduce body weight in rats that were fed HFD (Figure 4c). Moreover, the feeding behavior of these obese rats was reduced by using liraglutide, but not by SA (Figure 4d).

## 3. Discussion

Our work revealed that SA has the ability to stimulate GLP-1 receptors in both laboratory settings and in living organisms. This adequately explains SA’s antihyperglycemic effect in diabetic rats [20]. Liraglutide has shown efficacy in lowering plasma lipids [30]; thus, it contributes to the prevention of atherosclerosis and reduces the risk of unfavorable cardiovascular events [31]. SA, like the established GLP-1 receptor agonist liraglutide [32], may induce anti-inflammation and hyperlipidemia in animals. However, anti-obesity and anorexia appear to be less pronounced in SA-treated animals compared with liraglutide-treated animals.

SA is a phenolic aldehyde that has been found in a wide range of plants [29]. SA is mostly used as a food additive and as a natural redox mediator of fermentation. Interestingly, there is a substantial correlation between SA and the GLP-1 receptor [22]. Consequently, we conducted a scientific screening to validate this notion. In the current study, we found that the antihyperglycemic activity of SA was reduced when it was combined with the GLP-1 receptor antagonist Ex-9 [33]. Furthermore, following the outline of a previous study [34], we examined the immediate impact of SA on CHO-K1 cells that were genetically modified with the GLP-1 receptor gene. Only in the presence of the GLP-1 receptor gene in CHO-K1 cells did SA increase the intracellular cAMP concentration in a dose-dependent manner. It supported GLP-1 receptor mediation and the SA-induced influence in vitro. Furthermore, as previously described [35], we used Min-6 cells to support the direct effect of SA on pancreatic GLP-1 receptors using insulin secretion as an indicator. Furthermore, earlier investigations found that SA enhanced insulin secretion in pancreatic cells of diabetic mice in a dose-dependent manner in the presence of 15 mmol/L glucose [36]. Ex-9, interestingly, blocked insulin secretion at the dose required for the GLP-1 receptor blockade [16]. As a result, SA can activate the GLP-1 receptor to increase insulin secretion from pancreatic cells, which has not been previously mentioned. Like myricetin, which is another natural product may activate the GLP-1 receptor [37], SA promoted GLP-1 receptor mRNA levels in H9c2 cells in a dose-dependent manner. The current study expands on previous work concerning the direct effect of SA on the cardiac GLP-1 receptor in vitro. However, Western blots will be helpful to support this view in the future.

GLP-1 analogues have been shown to be anti-inflammatory [38]. SA also exhibits anti-inflammatory effects [22]. Plasma pro-inflammatory cytokines were widely applied as biomarkers because they were increased due to functional disorders, including hyperglycemia [23]. SA, like liraglutide, inhibited the increase in pro-inflammatory cytokines in diabetic rats’ plasma in a dose-dependent manner. Inflammation is a protective process that involves the immune system, the circulatory system, and molecular mediators. Macrophages are classified as pro-inflammatory (M1) or anti-inflammatory (M2) in a simplified model. M1 macrophages are activated by inflammatory cytokines, and they contribute to the ongoing inflammatory response by producing TNF-α and other proteins. IL-10 and IL-4/IL-13 stimulate M2 macrophages, which promote tissue repair and healing [24]. Notably, insulin resistance was associated with changes in macrophage phenotypes, from anti-inflammatory (M2 macrophages) to pro-inflammatory (M1 macrophages), in both mice and humans [39]. Liraglutide inhibits inflammation by substantially lowering plasma TNF-α, IL-1, and IL-6 levels [25]. SA inhibited plasma proinflammatory cytokines in the same way as liraglutide. Furthermore, SA, like liraglutide, increased the plasma levels of IL-10, which is an anti-inflammatory cytokine [40]. This viewpoint, however, was not included in the review article that introduced SA [22]. In animal models of Alzheimer’s disease, liraglutide has been shown to increase neurogenesis, improve cognitive function, and reduce amyloid plaque deposition based on its effect on M2 macrophages [41]. As a result, the effect of SA on neurodegeneration shall be studied in the future.

The current investigation demonstrates that SA has the potential to enhance lipid metabolism and ameliorate hepatic damage caused by diabetes. These results are comparable with those obtained using liraglutide in rats with type-2 diabetes [42]. Lipid metabolism is regulated by GLP-1, and liraglutide has been demonstrated to ameliorate hyperlipidemia [30]. The mechanism(s) for liraglutide remained obscure, which is likely due to the complicated parameters [43]. As with liraglutide, SA attenuated the plasma-increased lipids, including total cholesterol (TC) and triglycerides (TGs), in a dose-dependent manner. Therefore, SA has an ability to ameliorate lipid metabolism [44] in addition to the antioxidative effect [45]. However, details of the mechanisms regarding SA improved hyperlipidemia shall be investigated in advance.

Liver damage and nephropathy are recognized as common complications of T2DM [46]. The GLP-1 receptor is present on hepatocytes [47]. Therefore, GLP-1 may reduce the likelihood of non-alcoholic fatty liver disease (NAFLD) in individuals with T2DM by directly interacting with hepatic GLP-1 receptors. In accordance with the previous finding [48], we administered a low dose of STZ to HFD-fed rats to induce the mimic model. Diabetes was linked to a significant increase in blood ALT and AST levels, indicating damage to hepatocytes [49]. The current investigation shows that SA had a dose-dependent effect on restoring plasma AST and ALT levels. Furthermore, recent research has shown that cinchonine, an alkaloid derived from cinchona bark, effectively enhances non-alcoholic steatohepatitis (NASH) in laboratory mice [19]. This did not occur in mice that lacked GLP-1 receptors, which demonstrates the association between GLP-1 receptors and liver injury. 

Diabetic nephropathy is a common microvascular change that occurs during diabetic complications [33]. Additional research on this phenomenon is needed, with a special focus on the targets of molecular mechanisms and emerging therapies [34]. Diabetic rats showed a significant increase in renal function indicators, such as BUN and creatinine [50]. The current investigation demonstrated that SA exhibited a dose-dependent reversal of BUN and creatinine alterations, as with liraglutide. This suggests that SA has potential benefits in the treatment of diabetic nephropathy. Collectively, the administration of SA, as with the influence of liraglutide, may alleviate the impacts of hepatitis and diabetic nephropathy in diabetic rats [51,52]. 

It has been demonstrated that liraglutide reduces food consumption to improve obesity [53]. The results of the current report are completely consistent with these findings when using the diabetic model. In obese rats, however, SA was less effective than liraglutide in terms of weight control and food intake. This appears to be due to the complicated mechanisms of GLP-1 analogues during obesity regulation, such as the hippocampal GLP-1 receptor, which has been known to regulate food intake [54]. SA, on the other hand, may have neuroprotective effects against cerebral ischemia (MCAO)-induced injury [55]. It is likely that SA may affect the central nervous system. Moreover, recent studies have shown that fibroblast growth factor-21 (FGF21) is necessary for the weight reduction generated by liraglutide in mice that were given high-carbohydrate diets [56]. The effect of SA on FGF21 levels shall be studied further in the future. Tirzepatide, the first ‘twincretin’, was developed for obesity treatment; it works by acting as an agonist on both GLP-1 and GIP receptors [8]. It is important to highlight the limitations of the current study, particularly the minor impact of SA on GIP, since this seems to be an important factor in the observed outcome. Thus, more evidence is necessary to substantiate this perspective beforehand. Furthermore, a broader range of dosages should be explored to determine the most effective and safe dosage for SA. Additionally, it is important to investigate a comparison with other GLP-1 receptor agonists in experimental conditions to provide further information about the effectiveness of SA.

Recent studies demonstrate that some natural products, including geniposide [57] and myricetin [58], may activate the GLP-1 receptor to improve diabetes. Furthermore, it has been shown that morroniside functions as an agonist for GLP-1 receptors [18], and it has also been found to interact with sodium–glucose cotransporter 2 (SGLT2) [59]. Therefore, when using natural products in the treatment of diabetes, understanding their pleiotropic impact on the disease should be prioritized. 

## 4. Materials and Methods

### 4.1. Materials

Liraglutide was obtained from Novo Nordisk A/S (Bagsvaerd, Denmark). Exendin 9–39 (Ex9), SA (purity: 98%), and other chemicals or reagents were obtained from Sigma-Aldrich (St. Louis, MO, USA).

### 4.2. Animal Model

In this study, we deliberately caused diabetes in two distinct animal species by administering the condition to male Sprague Dawley (SD) rats obtained from the National Laboratory Animal Center in Taipei, Taiwan. In the T1DM model, rats weighing between 270 and 285 g, who had undergone overnight fasting, were administered an intravenous injection of STZ (Merck, Darmstadt, Germany) at a dosage of 65 mg/kg [26]. STZ was dissolved in sodium citrate buffer (pH 4.5) to a final concentration of 20 mg/mL [60]. Once the plasma glucose level in the rats treated with STZ reached a value higher than 300 mg/dL after one week, the rats were categorized as having type 1 diabetes. Subsequently, two weeks after the onset of diabetes, the experiments were conducted.

For the type 2-like diabetic model (T2DM), fructose was combined with an HFD to induce diabetes in younger rats weighing 150 to 180 g, as described previously [48]. Moreover, intraperitoneal injections of STZ, at low doses (35 mg/kg), were repeated every Monday for 6 weeks. Fructose (20%) in drinking water [61], combined with an HFD containing 34.9% (wt/wt) of fat (58Y1; Test Diet, Richmond, IN, USA), was applied to replace the rats’ regular diet (Rodent Laboratory Chow 5001, Purina, St. Louis, MO, USA). Tolbutamide (10 mg/kg, i.p.)-induced hypoglycemia was employed to confirm the success of induction in this model. A loss and/or marked reduction of responses indicated the presence of insulin resistance, and this occurred approximately 8 weeks later in all rats. Then, we used these animals in the T2DM for further experimentations in the current study.

The experimental approaches employed in animal-based research were conducted in accordance with the 1996 NIH Guide for the Care and Use of Laboratory Animals, and they were approved by Tajen University’s Local Ethics Commission for Animal Experiments (IACUC 111-07). The rats were administered sodium pentobarbital (35 mg/kg) intraperitoneally prior to the surgical procedures to alleviate animal distress.

### 4.3. Experimental Protocol

One cohort comprising eight rats was randomly selected to receive SA, which had been dissolved in normal saline, as a therapeutic intervention [62]. It has been noted that the oral intake (PO) of SA attenuates hyperglycemia [21]. Thus, in the current investigation, SA was delivered orally at a dosage that was shown to be efficacious. Furthermore, the acute effect of SA in rats was induced with a bolus injection of SA. To further understand the association between SA and GLP-1, animals received a pretreatment of Ex-9 30 min prior to the SA injection. Ex-9 were intraperitoneally delivered at the doses of 0.05 mg/kg and 0.1 mg/kg [63]. Simultaneously, liraglutide was delivered intraperitoneally (i.p.) as a positive reference. 

In rats with T1DM, diabetic rats were administered SA orally at two different dosages (5 mg/kg as a low dose and 20 mg/kg as a high dose) once daily. Another group received liraglutide (0.2 mg/kg, i.p.) to compare with SA. The results were compared with the control group which received the same volume of vehicle. Plasma cytokine levels were measured by obtaining blood samples from the tail veins of fasting diabetic rats, while they were under anesthesia, during a 4-week period of chronic treatment. We then analyzed the disparities between the control group treated with the vehicle and the group experiencing chronic effects. 

The long-term effects of liraglutide and SA were investigated using an eight-week therapy regimen in diabetic rats. Blood samples were collected while the subject was under anesthesia, namely from the tail vein, to quantify the concentrations of plasma biomarkers that were examined later. We then assessed the disparities between the treated samples and the control group treated with the vehicle. Following the treatment, blood samples were collected from the tail veins of rats under anesthesia. 

### 4.4. Laboratory Determinations

The plasma glucose level was tested using an analyzer (Quik-Lab, Ames; Miles Inc., Elkhart, IN, USA) via the glucose oxidase approach, as mentioned in our previous paper [64].

TNF-α, IL-6, and IL-1β are pro-inflammatory cytokines, while IL-10 is anti-inflammatory. Their plasma levels were quantified using a commercially purchased enzyme-linked immunosorbent assay (ELISA) kit from Sigma Aldrich (St. Louis, MO, USA). The other plasma levels of lipid profiles, such as total cholesterol (TC) and triglycerides (TGs), were tested using commercial kits from Cayman Chemical, located in Ann Arbor, MI, USA. The levels of creatinine and blood urea nitrogen (BUN) were measured using enzymatic techniques provided by Hoffman-La Roche Ltd., based in Basel, Switzerland. The liver enzymes, alanine aminotransferase (ALT) or aspartate aminotransferase (AST), were quantified using an automated analyzer and a commercially available kit (Roche, Germany).

The body weight and food consumption of rats with type 2 diabetes were measured after the long-term administration of either SA or liraglutide. The diabetic rats were given 200 g of the regular laboratory chow (pellet diet), and they were permitted to eat freely for one day, following a similar method to that used in a previous work [32]. A quantification of daily food consumption was conducted. The body weight of each rat was assessed and documented every two weeks.

### 4.5. The Cultured Cells

The current work used three types of cell lines for experimentation, namely CHO-K1 cells (CHO Cells), pancreatic Min-6 cells (Min-6 Cells), and cardiomyocytes (H9c2 Cells). The specimens were acquired from the Culture Collection and Research Center of the Food Industry Institute (CCRC) and cultivated for the specified treatment, following the procedure outlined below.

#### 4.5.1. CHO-K1 Cells

The human GLP-1 receptor gene was introduced into CHO-K1 cells via transfection, following the methodology described in a recent study [35]. The effective introduction of genetic material was verified the next day using the quantitative polymerase chain reaction (qPCR) technique outlined below. Cells expressing GLP-1 receptors were then exposed to SA at the specified doses for 1 h. The intracellular cAMP levels were then assessed with a commercially available ELISA kit (ADI-900-067, Enzo Life Sciences, Farmingdale, NY, USA). The specified samples were subjected to triplicate assays. A control group of CHO-K1 cells that had not been transfected with the GLP-1 receptor gene was used. The cAMP concentrations of different groups were compared.

#### 4.5.2. Min-6 Cells

Min-6 cells were cultured using an F-12K growth medium supplemented with 10% fetal bovine serum, as described previously [36]. The cells were propagated using trypsin (GIBCO-BRL Life Technologies, Gaithersburg, MD, USA) every 3 days, while the culture medium was refreshed every 2–3 days. In order to determine the specific impact of SA on insulin production from pancreatic cells via GLP-1 receptors, Min-6 cells were cultured in 12-well plates, with 1 mL of DMEM, 24 h before the experiment. Then, Min-6 cells were pretreated for 30 min in a KRBH buffer containing 15 mmol/L glucose with or without the antagonist at the specified concentrations. The cells were then placed in a solution of SA at the specified concentration for 1 h. After collecting the liquid component of the samples, the insulin concentrations in the liquid were quantified using an insulin ELISA kit (Mercodia, Uppsala, Sweden). Each measurement was conducted twice.

#### 4.5.3. H9c2 Cells

H9c2 cells were grown in accordance with the method reported in reference [37]. In summary, the H9c2 cells were cultured in Dulbecco’s modified Eagle’s medium (DMEM, pH 7.2; Gibco-BRL Life Technologies, Gaithersburg, MD, USA) with 10% fetal bovine serum. The cells were seeded at a density of 6000 cells per square centimeter, and underwent proliferation in the culture medium. The medium was substituted on the second day. The cells were used for future tests, because H9c2 cells exhibit comparable responses to primary rat neonatal cardiomyocytes [37]. The mRNA levels in H9c2 cells were measured and the results were used to detect the changes in GLP-1 receptor in these samples.

### 4.6. Quantitative Reverse-Transcription Polymerase Chain Reaction (qRT-PCR)

TRIzol was used to separate the total RNA from H9c2 cells. The qRT-PCR primers were acquired from Roche (Roche Diagnostics GmbH, Mannheim, Germany). The concentration of each PCR product was assessed by comparing it with a matched standard curve. The analysis of relative gene expression data was conducted using real-time quantitative PCR and the 2-ΔΔCq technique [38]. β-actin was used as an internal control for normalizing gene expression [23]. The experiment was independently performed three times. The primers used were as follows:

GLP-1 Receptor:
forward: 5′-AGTGCGAAGAGTCCAAGCAA-3′
reverse: 5′-TTGAGGGCAGCGTCTTTGAT-3′

β-Actin:
forward: 5′-CATCCAGGCTGTGTTGTCCC-3′
reverse: 5′-CACGCACGATTTCCCTCTCA-3′

### 4.7. Statistical Analysis

The findings are shown as the average plus the standard error of the mean (SEM) for each group, according to the specified sample size. The data were subjected to a two-way analysis of variance using SPSS statistical software version 21 for analysis. S Dunnett’s post hoc analysis was performed on the data (SPSS Inc., Chicago, IL, USA). The statistical significance was defined as *p* < 0.05.

## 5. Conclusions

The findings clearly show that SA effectively alleviated diabetes symptoms in two different animal models by activating the GLP-1 receptor. This novel perspective has not been previously discussed, except a study which concerned the docking. SA may also stimulate the release of insulin directly from the pancreatic beta-cell, which expresses the GLP-1 receptor. Hence, SA exhibits the capacity to serve as a prospective anti-diabetic drug in the future.

## Data Availability

The data presented in this study are available from the corresponding author upon reasonable request.
